# Validity and reliability of “*My Jump app*” to assess vertical jump performance: a meta-analytic review

**DOI:** 10.1038/s41598-023-46935-x

**Published:** 2023-11-17

**Authors:** Cebrail Gençoğlu, Süleyman Ulupınar, Serhat Özbay, Murat Turan, Buğra Çağatay Savaş, Selim Asan, İzzet İnce

**Affiliations:** 1 Faculty of Sport Sciences, Erzurum Technical University, Erzurum, Turkey; 2 Faculty of Sport Sciences, Ankara Yıldırım Beyazıt University, Ankara, Turkey

**Keywords:** Computational biology and bioinformatics, Public health

## Abstract

This systematic review and meta-analysis aims to investigate the validity and reliability of the My Jump smartphone application in measuring vertical jump height, specifically using flight-time-based measures. To identify potential studies for inclusion, a comprehensive search strategy was employed in PubMed, Web of Science, Scopus, and EBSCO host databases. Validity was assessed in two ways: (1) mean and standard deviations of My Jump measurements were compared to criterion methods to assess the agreement of raw scores; (2) correlation coefficients evaluated the within-group consistency of rankings between My Jump and criterion methods. Reliability was assessed using intraclass correlation coefficients (ICC). Heterogeneity was evaluated via Cochrane’s Q statistic, its p-value, I^2^ value, and tau^2^ value. Publication bias was explored through funnel plot symmetry and confirmed with extended Egger’s test. Following the search, 21 studies met the inclusion criteria. Results showed no significant difference in raw scores between My Jump and criterion methods, indicating high agreement. High correlation was also found for within-group rankings, suggesting consistency. The My Jump application demonstrated nearly perfect reliability scores. The My Jump application appears to be a valid and reliable tool for sports scientists and strength and conditioning practitioners, offering a cost-effective and accessible means for accurately assessing vertical jump performance in various settings. However, it should be noted that these results are specific to flight-time-based measures, and further research is needed to validate these findings against gold-standard take-off velocity methods.

## Introduction

The emergence of novel devices (e.g. *My Jump* smartphone application, GymAware, PUSH Band) measuring athletic performance is quickly gaining momentum as these devices increase in popularity as potential alternatives to expensive laboratory equipment^1,2^. Their main advantage is that these novel devices are easily portable (especially in the case of software applications that are integrated into tablets and smartphones); they have the potential to offer an excellent solution to the problems of many laboratory-based measurement methods such as the high cost of laboratory equipment, the difficulties of transporting the devices to the field, or people to the laboratory, and the need for periodic maintenance and complex interfaces^2–4^. However, to take advantage of all these facilitating aspects, it is necessary to ensure that the measurements made with these methods give valid and reliable outputs.

Validity of the measurement of athletic characteristics requires that the movement pattern is close to the mode and profile observed during competition, that there is an output that represents specific proficiency, that the measurements are associated with a proven gold standard or a criterion measurement, that the evaluations can predict the actual competition performance, and that the results can distinguish between the successful and unsuccessful athletes^5–7^. Similarly, reliability of the measurement of athletic characteristics requires that the measurements can be replicated, that the within-group ranking and agreement between the raw scores can be maintained from test to retest, and that successive measurements give the same output when performed in such a short time that no actual performance improvement is possible^8–10^. Hence, a novel device or method designed to measure a component of physical fitness is expected to meet all these requirements regarding validity and reliability.

The need to easily measure, evaluate, and monitor athletic performance has inspired sports engineering professionals to design or produce novel smartphone applications^1,2^. Mobile applications have become very popular due to many beneficial features such as being portable, inexpensive, and easily accessible. For example, the *My Jump* smartphone application based on high-speed video technology provides an extremely practical way of measuring vertical jump performance and has been used in numerous scientific studies since its introduction to the literature^11–13^. However, although there are many advantages provided by *My Jump*, there are several potential sources of bias that may impact the measurement of jump height. These include the need to manually identify the take-off and landing frames of the video, the potential for knee and ankle flexion (dorsiflexion) just before landing to artificially extend the measured flight time, the possibility of one foot making contact with the ground before the other, which could be misleadingly used as the frame to determine the end of the flight time, and the consideration that a slight contact by one foot may not accurately represent the displacement of the center of mass^14–16^. Therefore, sports scientists, conditioners, coaches, and athletes need to be confident that this smartphone application can present consistent and repeatable outputs.

In the realm of jump height calculations, two distinct definitions based on the Centre of Mass (COM) displacement are commonly used^16,17^. The first measures the COM displacement from the initial flat-footed standing position to the apex of the jump^18^. The second focuses on the COM displacement from the moment of take-off to the jump’s peak^16,17^. The My Jump smartphone application predominantly employs the latter method, offering greater flexibility in measuring jump performance, as it does not require a specific starting position^3,19^. While traditional Force Platforms and 3D motion capture systems are considered the ‘gold standard’ for jump height measurement due to their high levels of accuracy and reliability^16,20^, they come with challenges such as high costs, complex setup procedures, and limited portability^1,3,19^. Hence, practitioners frequently turn to more cost-effective and portable alternatives like My Jump^2,13,21^. However, users should be aware that the application comes with potential sources of error. These include the risk of artificially extending the flight time due to knee and ankle flexion (dorsiflexion) just before landing, among other factors.

The calculation methods for jump height are generally categorized into two main groups: Indirect and Direct methods^16,21^. Indirect methods, such as the Flight Time Method, Impulse-Momentum Method, and Double Integration Method, involve complex mathematical calculations and are often used in conjunction with force platforms to provide vertical ground reaction force data^16,17,19,22^. Direct methods, on the other hand, provide jump height values directly from vertical jump systems or are derived from motion capture systems^16,23,24^. The choice of calculation method and equipment can significantly impact the reliability, validity, and accuracy of the jump height measurements^2,3,5^. In this context, the Impulse-Momentum Theorem provides a reliable and objective measurement methodology^15^. This theorem is logically equivalent to Newton’s second law of motion, stating “Impulse (I) is equal to mass (m) times velocity (v)”. Force plates calculate jump height using this velocity value. Specifically, the square of the take-off velocity is divided by 9.81, and this result is then halved. These calculations are based on the Law of Conservation of Energy and yield highly reliable and valid jump height values. Therefore, the Impulse-Momentum Theorem offers an effective method for accurately measuring vertical jump height and minimizes the possibilities of ‘gaming the system’ or manipulation in competitive settings^16^. However, Due to their intricate configurations and substantial costs, traditional methods like force platforms are often unsuitable for field use, paving the way for the increasing popularity of My Jump smartphone application that offer instant results without the need for complex setups or specialized training. While they may lack the capability to provide vertical ground reaction force data, their user-friendly interfaces and instant feedback features make them highly practical for field use^3,16,25^.

The vertical jump is a dynamically appropriate movement pattern as the movement mechanics required in the actual competition can be repeated during tests and training^26,27^. As a result of all these positive aspects, jump tests have become indispensable for globally used test batteries. These include EUROFIT, a European test battery for assessing physical fitness; FITNESSGRAM, an American health-related physical fitness assessment; and ALPHA, a test battery designed to assess the lifestyle and fitness of young people^28–30^. The *My jump* is the most popular technology developed to measure jump performance in the field of sports sciences^2,3,19^. There is a consensus that the *My Jump* provides many benefits, such as being user-friendly, accessible, portable, and affordable. Some studies report that this smartphone application yields valid and reliable results, whereas there are also concerns that the smartphone application may produce somewhat doubtable results^1,2,14^. Therefore, there is a necessity for a pooled evaluation of the research findings reporting the validity and reliability of the *My Jump* smartphone application in different populations. This study, which is composed of a systematic review and meta-analysis, aims to investigate whether the *My Jump* smartphone application (versions 1 and 2) produces valid and reliable results when measuring vertical jump height.

## Methods

This systematic review and meta-analysis was conducted following the guidelines of the Cochrane handbook^31^ and following the recommendations of the Preferred Reporting Items for Systematic Reviews and Meta-Analyses (PRISMA)^32^. We registered in the PROSPERO database (International Prospective Register of Systematic Reviews) with reference number CRD42022295759.

### Data sources and search strategy

To identify potential studies for inclusion in this systematic review and meta-analysis, a search strategy was implemented in PubMed, Web of Science, Scopus, and EBSCO host databases from the date of their inceptions through to the 31st of September 2023. The search term was set to “My Jump” AND (“Validity” OR “Reliability”). Additionally, reference lists of the included studies, previous systematic reviews, and meta-analyses were reviewed for any other relevant studies. The search was limited to the English language. Three authors (CG, MT and SU) screened the titles and abstracts, and articles with the potential to be included in the study were read in full for further examination. Relevant studies were assessed against the inclusion and exclusion criteria described below. Inter-author disagreements were resolved by consensus decisions or by the senior author's final decision (İİ).

### Inclusion criteria

The inclusion criteria for the articles in the systematic review and meta-analysis were focused on the types of studies, testing methods, participants, variables, statistical analysis, and reported outputs. To be included, the studies had to: (1) be original research; (2) be published in a peer-reviewed scientific journal; (3) have the full text available in English; (4) investigate vertical jump performance; (5) have included human participants such as athletes, untrained individuals, adults, elderly, children, etc.; (6) have investigated validity or reliability scores of the *My Jump* app; (7) report the Pearson r correlation, regression, and intraclass correlation coefficients (ICCs), or means and standard deviations.

### Exclusion criteria

The following types of studies were excluded from the present meta-analysis: (1) studies in a language other than English; (2) unpublished studies, reviews, book chapters, editorials, non-peer-reviewed texts, case studies, abstracts, theses; (3) studies not reporting any validity or reliability statistics; (4) studies that focused on animal experiments; (5) studies using an application other than *My Jump*; (6) studies examining a physical fitness characteristic other than the vertical jump; (7) studies examining the effect of an exercise intervention on vertical performance.

### Data extraction

The extracted data included the authors, year of publication, sample subject characteristics (age, body mass, height), criterion, type of vertical jump, means and standard deviations of the *My Jump* and criterion measurements, and the validity and reliability outputs (Table 1). Three authors (CG, MT and SÖ) independently extracted the data from the selected articles using a pre-defined form created in Microsoft Excel (Microsoft Corporation, Redmond, WA, USA). If there were any disagreements between the authors about the extracted data, the accuracy of the information was re-checked to reach a consensus.

**Table 1:** Descriptive information of included studies.

Study	Sample characteristics	Criterion	Activity patterns	Study design	My Jump results (mean ± SD)	Criterion results (mean ± SD)	Validity outputs	Reliability outputs
Alias et al. (2021)	N = 25, recreational athletes	None	CMJ	Participants performed CMJ for five attempts and was recorded by using an iPhone 7 Plus in 240 frames per second. The videos were then rated by three raters and rated again 7 days later using the My Jump 2 mobile application	23.7 ± 5.7	None	None	ICC = 1.00 (inter-rater) ICC = 1.00 (intra-rater) ICC = 1.00 (within session-device)
Balsalobre-Fernández et al. (2015)	N = 20, recreationally active, healthy, sport sciences male students, age = 22.1 ± 3.6 years, height = 181 ± 8 cm, body mass = 74.0 ± 10.4 kg	Force platform (Kistler 9287 BA, Kistler Instruments Ltd., Hook, UK)	CMJ	Participants performed five CMJs on the force platform while being recorded with an iPhone 5 s. Each jump was separated by a 2-min passive rest period	Not reported	Not reported	Pearson r = 0.995	ICC = 0.997 (within session-device); 0.999 (inter-rater)
Barbalho et al. (2021)	N = 11, male soccer players, age = 18.2 ± 1.3 years, height = 174 ± 7 cm, body mass = 69.9 ± 9.5 kg	Force platform (BIOMEC400; EMG System do Brasil, Ltda, SP, Brazil)	DJ30	The data collection was done in a single session. The My Jump 2 app and force plate data were recorded simultaneously from the 3 jumps that were performed by each athlete	33.1 ± 11.5	33.4 ± 11.6	Not reported	ICC = 1.00 (inter-rater) ICC = 1.00 (intra-rater)
Bishop et al. (2022a)	N = 27, postgraduate sport science students, age = 26.3 ± 5.1 years, height = 178 ± 6 cm, body mass = 82.8 ± 11.7 kg	Force platform (Hawkin Dynamics, Westbrook, ME, USA)	CMJ	Participants performed three practice trials of the CMJ, at their perceived maximal effort. An iPad device was mounted to a tripod at a height of 0.75 m at a distance of 3 m from the front of the force plates	37.0 ± 8.0	37.0 ± 7.0	Pearson r = 0.98	ICC = 0.997 (within session-device)
Bishop et al. (2022b)	N = 30, national-level youth basketball athletes, age = 17.7 ± 1.3 years, height = 181 ± 10 cm, body mass = 73.3 ± 13.3 kg	None	CMJSL DJSL	Participants performed single-leg countermovement jumps and single-leg drop jumps all assessed using the My Jump 2 app. To analyze the jump tests, a trained sports scientist with 2 years of experience in slow-motion video apps recorded a video of each test for its analysis using the My Jump 2	CMJSL_L1 = 13.7 ± 5.4 CMJSL_L2 = 13.7 ± 5.9 CMJSL_R1 = 14.2 ± 5.3 CMJSL_R1 = 13.3 ± 4.7 DJSL_L1 = 11.6 ± 6.0 DJSL_L2 = 11.0 ± 5.7 DJSL_R1 = 12.1 ± 5.9 DJSL_R1 = 11.3 ± 5.7	None	None	ICC = 0.965 (within session-device for CMJSL_L); 0.940 (within session-device for CMJSL_R) ICC = 0.975 (within session-device for DJSL_L); 0.965 (within session-device for DJSL_R)
Bogataj et al. (2020a)	N = 44, recreationally active and had membership in the local gym in Subotica, Serbia, 26 male (age = 30 ± 10 years, height = 178 ± 16 cm, body mass = 86 ± 24 kg) and 18 female (age = 29 ± 5 years, height = 170 ± 6 cm, body mass = 60 ± 9 kg)	Photocell system (Optojump photocell system; Microgate, Bolzano, Italy)	CMJ CMJAS SQJ	Participants performed three jumps (in three styles) on the photocell system while being recorded with an iPhone X. Each jump was separated by a 2 min rest period	CMJ: 31.9 ± 6.6 SQJ: 29.6 ± 6.0 CMJAS: 39.4 ± 9.7	CMJ: 32.5 ± 7.1 SQJ: 30.0 ± 6.3 CMJAS: 39.7 ± 9.5	CMJ, Pearson r = 0.98 (for males); 0.96 (for females) Squat Jump, Pearson r = 0.95 (for males); 0.97 (for females) CMJ with arm swing, Pearson r = 0.98 (for males); 0.94 (for females)	CMJ, ICC = 0.96 (absolute agreement for males); 0.97 (absolute agreement for females) Squat Jump, ICC = 0.93 (absolute agreement for males); 0.94 (absolute agreement for females) CMJ with arm swing, ICC = 0.97 (absolute agreement for males); 0.97 (absolute agreement for females)
Bogataj et al. (2020b)	N = 48, primary school children from Subotica, Serbia, 26 male (age = 12.3 ± 0.8 years, height = 159 ± 13 cm, body mass = 51.8 ± 18.3 kg) and 22 female (age = 11.8 ± 0.8 years, height = 157 ± 10 cm, body mass = 50.6 ± 11.6 kg)	Photocell system (Optojump photocell system; Microgate, Bolzano, Italy)	CMJ CMJAS SQJ	Participants performed three jumps (in three styles) on the photocell system while being recorded with an iPhone X. Each jump was separated by a 2 min rest period	CMJ: 24.5 ± 4.7 SQJ: 22.3 ± 4.1 CMJAS: 27.0 ± 5.8	CMJ: 24.6 ± 4.3 SQJ: 22.2 ± 4.5 CMJAS: 27.2 ± 5.8	CMJ, Pearson r = 0.97 Squat Jump, Pearson r = 0.97 CMJ with arm swing, Pearson r = 0.99	CMJ, ICC = 0.96 (absolute agreement) Squat Jump, ICC = 0.88 (absolute agreement) CMJ with arm swing, ICC = 0.93 (absolute agreement)
Brooks et al. (2018)	N = 26 (14 male and 12 female), healthy and recreationally active adults, age = 23.2 ± 3.5 years, height = 170 ± 10 cm, body mass = 74.0 ± 10.4 kg	(1) AMTI AccuPower force platform (Advanced Mechanical Technology Inc., MA, USA) (2) Yardstick jump-and-reach apparatus	CMJAS	Participants completed the test protocol on two occasions, separated by a minimum of seven days. Participants performed three jumps on the force platform while being recorded with an iPhone 5 s. Each jump was separated by a 2 min passive rest period	Not reported	Not reported	Pearson r = 0.98 (Force platform); 0.94 (Yardstick)	ICC = 0.97 (inter-session); 0.99 (intra-rater)
Carlos-Vivas et al. (2018)	N = 40 (29 male and 11 female), recreationally active, healthy, sport sciences students, age = 21.4 ± 1.9 years, height = 174 ± 7 cm, body mass = 68.7 ± 8.4 kg	Force platform (BioWare v. 5.3.0.7, Kistler holding aG, Winterthur, Switzerland)	CMJ	Participants performed five CMJs on the force platform while being recorded with an iPhone 6	28.6 ± 7.2	28.7 ± 7.2 (time in the air method) 28.4 ± 6.8 (velocity at takeoff method)	ICC = 1.00 (inter-instruments reliability for the time in the air method); 0.996 (inter-instruments reliability for the velocity at takeoff method2);	ICC = 0.983 (within session-device)
Chow et al. (2023)	N = 30, physically active college students, 14 males and 16 females, age = 23.0 ± 1.7 years, height = 168 ± 6 cm, body mass = 61.9 ± 9.8 kg	Takei Vertical Jump Meter (Takei Scientific Instruments Co., Niigata, Japan), HomeCourt (homecourt.ai)	CMJ	Participants visited the laboratory twice, with two days in between, and performed three jumps each day. All jumps were recorded by My Jump 2, HomeCourt, and the Takei Vertical Jump Meter simultaneously	40.9 ± 7.9	46.1 ± 7.6 (HomeCourt) 42.0 ± 8.1 (Takei Vertical Jump Meter)	Pearson r = 0.85 (My Jump 2—HomeCourt) Pearson r = 0.93 (My Jump 2—Takei Vertical Jump Meter)	ICC = 0.86 (within session-device for day 1) ICC = 0.88 (within session-device for day 2)
Cruvinel-Cabral et al. (2018)	N = 41, elderly people, 12 male (age: 73.2 ± 6.4 years, body mass = 68.3 ± 12.7 kg) and 29 female (age: 69.4 ± 8.9 years, body mass = 64.7 ± 12.6 kg)	Contact mat (Chronojump, version. 1.6.2; Boscosystem, Barcelona, Spain)	CMJ	Participants performed three CMJs. Contact mat and My Jump, were used simultaneously to assess VJ height	10.2 ± 5.1	10.01 ± 5.0	Pearson r = 0.999	ICC = 0.948 (within session-device)
Driller et al. (2017)	N = 61 (30 male and 31 female), recreational to highly trained athletes, age = 20 ± 4 years, body mass = 76.4 ± 15.2 kg	Force plate (Dual-Axis Force Platform, PASCO, California, USA)	CMJ	Participants performed three CMJs on the force platform while being recorded with an iPhone 6 s. Each jump was separated by 5 s	25.9 ± 7.9	25.1 ± 7.5	Pearson r = 0.96	ICC = 0.97 (inter-rater)
Gallardo-Fuentes et al. (2016)	N = 21 (14 male and 7 female), track and field national and international-level competitive athletes, age = 22.1 ± 3.6 years, height = 181 ± 8 cm, body mass = 74.0 ± 10.4 kg	Contact platform (Ergotester, Globus, Codogne, Italy)	CMJ SQJ DJ40	Participants completed the test protocol on two occasions, separated by 48 h. Participants performed five jumps in three styles on the contact platform while being recorded with an iPhone 5 s. Each jump was separated by a 2-min passive rest period	Test results: CMJ: 37.0 ± 10.3 SQJ: 35.7 ± 9.1 DJ40: 31.6 ± 5.9 Re-test results: CMJ: 36.8 ± 9.5 SQJ: 34.6 ± 9.1 DJ40: 31.6 ± 7.0	Test results: CMJ: 37.1 ± 9.9 SQJ: 35.8 ± 8.8 DJ40: 31.5 ± 5.9 Re-test results: CMJ: 36.8 ± 9.3 SQJ: 34.9 ± 8.6 DJ40: 31.7 ± 7.0	CMJ, Pearson r = 0.99 (between two instruments, first day); 0.99 (between two instruments, second day) Squat Jump, Pearson r = 0.99 (between two instruments, first day); 0.99 (between two instruments, second day) Drop Jump, Pearson r = 0.99 (between two instruments, first day); 0.99 (between two instruments, second day)	CMJ, ICC = 0.99 (within session-device for the first day); 0,99 (within session-device for the second day) Pearson r = 0.95 (inter-session) Squat Jump, ICC = 0.99 (within session-device for the first day); 0,98 (within session-device for the second day) Pearson r = 0.90 (inter-session) Drop Jump, ICC = 0.99 (within session-device for the first day); 0,98 (within session-device for the second day) Pearson r = 0.87 (inter-session)
Gür and Ayan (2023)	N = 24 (13 male and 11 female), healthy sedentary individuals, age = 22.3 ± 1.1 years, height = 170 ± 9 cm, body mass = 64.0 ± 10.7 kg	Smart Jump (Fusion Sport, Queensland, Australia)	CMJ	After the aerobic and dynamic stretching warm-up protocol, each participant performed four CMJ jumps with maximal effort. Participants were given a 2-min passive recovery between each trial. Jumps were recorded simultaneously with the My Jump 2 mobile app and Fusion Sport brand Smart Jump splash mat device	33.3 ± 7.3 (jump 1) 33.9 ± 7.0 (jump 2) 33.6 ± 6.4 (jump 3) 33.9 ± 6.2 (jump 4)	33.5 ± 7.3 (jump 1) 33.4 ± 6.9 (jump 2) 34.2 ± 6.2 (jump 3) 34.5 ± 6.2 (jump 4)	Pearson r = 0.99	ICC = 0.99 (within session-device)
Haynes et al. (2019)	N = 14, male sports sciences student, age = 29.5 ± 9.9 years, height = 178 ± 10 cm, body mass = 81.4 ± 14.1 kg	Force platform (FP8, Hurlab, Finland)	DJ20 DJ40	Participants completed the test protocol on two occasions, separated by seven days. Participants performed three jumps (drop heights of 20 and 40 cm) on the force plate while being recorded with an iPhone 5 s. Each jump was separated by a 2 min passive rest period	DJ_20_: 23.8 ± 7.3 DJ_40_: 22.6 ± 5.6	DJ_20_: 23.3 ± 6.2 DJ_40_: 23.3 ± 5.3	Drop jump from 20 cm, Pearson r = 0.812 Drop jump from 40 cm, Pearson r = 0.959	Drop jump from 20 cm ICC = 0.803 (within session-device) Drop jump from 40 cm ICC = 0.958 (within session-device)
Jimenez-Olmedo et al. (2022)	N = 39, active adult athletes, 25 male (age = 22.2 ± 2.7 years, height = 180.1 ± 4.4 cm, body mass = 77.6 ± 6.8 kg) and 14 female (age = 23.2 ± 1.8 years, height = 170.7 ± 4.4 cm, body mass = 66.2 ± 4.0 kg)	two iPhone 7 units (Apple Inc., Cupertino, CA, USA)	CMJ	Two identical smartphones recorded 195 countermovement jump executions at heights 30 and 90 cm, which were randomly assessed by three experienced observers. The videos were randomly analyzed in regard to the observation heights, jump trials, and participants	Not reported	Not reported	Not reported	CMJ, ICC = 0.99 (between-observer) ICC = 0.99 (within-observer)
Patiño-Palma et al. (2022)	N = 119, high-performance athletes from different sports disciplines, age = 18.5 ± 1.3 years, height = 174 ± 6 cm, body mass = 67.4 ± 6.0 kg	Chronojump Boscosystem (Barcelona, Spain), OptoGait (Bolzano, Italy), and Wheeler Jump (Wheeler Sports Tech, FL, USA)	CMJ	Jump performance was evaluated through the CMJ in a training session using the Chronojump Boscosystem contact platform, the OptoGait photoelectric system, and the My Jump 2 mobile application as measurement tools, comparing the results with the values obtained with the Wheeler Jump sensor	CMJ: 42.8 ± 6.9	CMJ: 39.3 ± 7.1	Rho = 0.994	ICC = 0.993 (within session-device)
Plakoutsis et al. (2023)	N = 34, 22 male and 12 female, collegiate athletes, age = 21.6 ± 5.7 years	KForce Plates (K-Invent, Montpellier, France)	CMJ	Participants performed three maximal CMJs while standing on a portable force platform. The jumps were recorded with a portable KForce plates system and My Jump 2 through iPhone 13 at the same time. Each participant repeated the testing procedure after seven days in order to assess the reliability of the measurements (ICC)	Not reported	Not reported	Pearson r = 1.00	Not reported
Soares et al. (2023)	N = 21 (15 male and 6 female), healthy, judo athletes, age = 26.4 ± 5.4 years, height = 172 ± 8 cm, body mass = 72.6 ± 12.9 kg	Chronojump Boscosystem (Barcelona, Spain)	CMJ	Participants performed two countermovement jumps on the Chronojump platform (42 jumps). Simultaneously, the videos of the jumps were captured using recommendations in the app and were later processed and analyzed independently by two evaluators	CMJ: 26.4 ± 8.9 (Evaluator 1) CMJ: 26.3 ± 8.1 (Evaluator 2)	CMJ: 26.7 ± 8.1	ICC = 0.94 (Evaluator 1 vs Chronojump) ICC = 0.97 (Evaluator 2 vs Chronojump)	ICC = 0.95 (inter-rater)
Stanton et al. (2017)	N = 29 (10 male and 19 female), healthy, recreationally active adults, age = 26.4 ± 5.4 years, height = 172 ± 8 cm, body mass = 72.6 ± 12.9 kg	AMTI BP400 800–2000 force plate (Advanced Mechanical Technology Inc., Watertown, MA)	CMJ DJ30	Participants completed the test protocol on two occasions, separated by seven days. Participants performed three jumps in two styles on the force plate while being recorded with an iPhone 5 s. Each jump was separated by a 2-min passive rest period	CMJ: 20.6 ± 8.5 DJ_30_: 19.4 ± 8.4	CMJ: 20.4 ± 7.6 DJ_30_: 20.3 ± 8.3	Pearson r = 0.997 Pearson r = 0.998	CMJ, ICC = 0.99 (within session-device); 0.99 (intra-rater) Drop Jump, ICC = 0.99 (within session-device); 0.99 (intra-rater)
Yingling et al. (2018)	N = 135, healthy adults, 94 male (age: 18–29 years, height = 177 ± 8 cm, body mass = 72.8 ± 9.9 kg) and 41 female (age: 18–39 years, height = 167 ± 8 cm, body mass = 63.5 ± 9.3 kg)	Vertec linear position transducers (JUMPUSA.com, Sunnyvale, CA, USA)	CMJAS	Participants performed three jumps. Vertec and My Jump, were used simultaneously to assess VJ height	43.05 ± 12.13	51.93 ± 14.36	Pearson r = 0.813	ICC = 0.813 (consistency); 0.665 (absolute agreement)

CMJ: countermovement jump; CMJSL: single leg countermovement jump; SQJ: squat jump; CMJAS: countermovement jump with arm swing; DJ: drop jump; DJSL: single leg drop jump; r: correlation coefficient; ICC: intraclass correlation coefficient.

### Methodological quality assessment

The methodological quality of each included study was assessed using a modified Downs and Black assessment scale^33^. A total of 8 domains were identified to evaluate the quality of reporting for studies included in this review: (1) the hypothesis/aim described; (2) whether the participants were representative of the target population; (3) the participant characteristics detailed; (4) the intervention procedure detailed; (5) the use of an appropriate reference test/criterion; (6) the use of appropriate statistical tests; (7) the main outcomes reported; (8) if the outcome measures valid and reliable. Each criterion was evaluated as low quality, moderate quality, high quality, inadequate, or unclear.

### Meta-analyses

Meta-analyses were conducted using comprehensive meta-analysis software, version 2 for Windows (CMA, Biostat company, Englewood, NJ, USA)^34^. The meta-analysis of validity was performed in two ways: (1) the means and standard deviations were compared between the *My Jump* and criterion measurements to assess the agreement of raw scores; (2) the correlation coefficients were used to determine the consistency of the rankings within-group in the *My Jump* and criterion measurements. Additionally, a meta-analysis of reported ICCs was performed to confirm reliability. However, the types of ICCs or Pearson r coefficients reported in the studies were varied (inter-rater, intra-rater, within-subject, between the devices, between two test days, between the consecutive jump performances of the same participant, etc.). Therefore, when a study reported multiple Pearson r coefficients or ICCs from the same sample group, the study was used as a single unit of analysis to avoid the overestimation of its contribution to the pooled result due to double counting. The pooled correlation values were interpreted according to a random-effects model in case of any heterogeneity between studies (when the *p*-value of the Q statistic was less than 0.1)^35^. For validity, pooled correlations were classified as follows: 0–0.19, “no significant correlation”; 0.2–0.39, “low correlation”; 0.4–0.59, “moderate correlation”; 0.6–0.79, “moderately high correlation”; and ≥ 0.8, “high correlation”^36,37^. The scale designed by Landis and Koch reliability strength thresholds was applied, as follows: 0.01–0.20, “mild reliability”; 0.21–0.40, “fair reliability”; 0.41–0.60, “moderate reliability”, 0.61–0.80, “substantial reliability”; and 0.81–1.00, “nearly perfect reliability”^38^. Sub-analyses were meticulously conducted to delve into the nuances of various factors affecting the outcomes. These sub-analyses were categorized based on three key parameters: the type of jump performed (CMJ, SQJ, and DJ), the criterion device used for measurement (Force Plates and other devices), and the type of reliability assessed (inter-rater and intra-rater reliability, inter-session, and within-session reliability).

Heterogeneity was determined by Cochrane’s Q statistic and its *p-*value, *I*-squared value, and *tau*-squared value^35,39,40^. The Q-value (and its *p*-value), which indicates whether all the studies have shared a common effect size, and the *I*-squared value, which refers to the proportion of the observed variance when the sampling error is eliminated (i.e. when observing the true effect size for all studies in the analysis), are the most common heterogeneity indicators^37,39,41,42^. The *I*-squared values of < 25%, 25–75%, and > 75% were considered to represent low, moderate, and high levels of heterogeneity, respectively^43^. The *Tau*-squared value is a measure of the variance of true effects representing a concrete and reliable heterogeneity^31,35,41^.

The risk of publication bias was explored using funnel plot symmetry, and asymmetries were confirmed using the extended Egger’s test^44^. Egger’s test regresses the standardized effect sizes on a measure of precision (i.e. standard errors of the correlation coefficients). A significant coefficient for Egger’s test means that the effect sizes and sampling variance for each study are related and indicates that a publication bias is present. In the case of evidence of a publication bias, Duval and Tweedie’s “trim and fill” procedure was applied to determine whether estimates required adjustment based on missing studies^37^. Additionally, sensitivity analyses were conducted by removing a study^14^ with validity concerns to assess the robustness of the pooled estimates.

## Results

### Study selection

**Figure 1:**
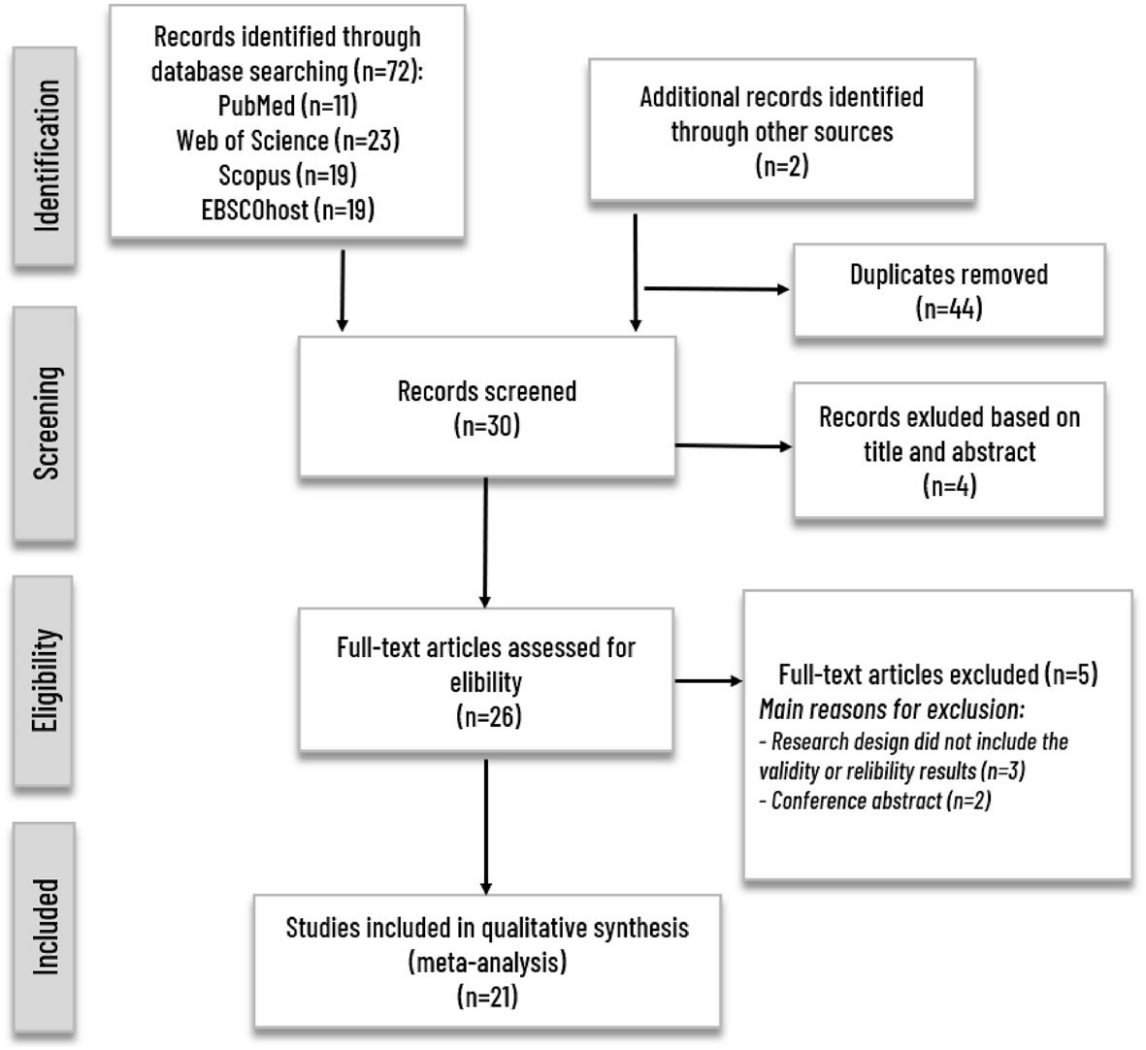
Flow chart of the review process.

We initially found a total of 74 potential research articles related to the *My Jump* smartphone application published until September 2023. After excluding the 44 duplicates and 4 studies based on their titles and abstracts, 26 studies were reviewed as full texts. Following the identification of studies meeting the inclusion criteria of this paper, a total of 21 studies consisting of 839 accumulated participants were included in the present meta-analysis (Fig. 1).

### Methodological quality

Using a modified Downs and Black assessment scale^33^, eight risk domains for the 21 individual research articles (a total of 168 scores) were evaluated. There were only nine items scored as low quality and one item scored as inadequate, while all other items were rated as moderate or high quality; therefore, the overall methodological quality was considered as moderate-to-high (Table 2).

**Table 2:** Methodological quality assessments of original studies included in meta-analyses.

Studies/items	1	2	3	4	5	6	7	8
Alias et al. (2021)	L	M	L	M	M	M	M	M
Balsalobre-Fernández et al. (2015)	H	L	H	M	H	H	L	M
Barbalho et al. (2021)	H	L	H	M	H	M	M	M
Bishop et al. (2022a)	H	M	H	M	H	H	M	M
Bishop et al. (2022b)	H	M	H	M	M	H	M	M
Bogataj et al. (2020a)	H	M	H	M	M	M	M	M
Bogataj et al. (2020b)	H	M	H	M	M	M	M	M
Brooks et al. (2018)	H	L	H	M	H	H	L	M
Carlos-Vivas et al. (2018)	H	M	H	M	H	M	M	M
Chow et al. (2023)	H	M	H	M	M	H	M	M
Cruvinel-Cabral et al. (2018)	H	M	M	M	M	M	M	M
Driller et al. (2017)	I	H	H	M	H	M	M	M
Gallardo-Fuentes et al. (2016)	H	L	H	M	M	H	M	M
Gür and Ayan (2023)	M	M	H	M	M	M	M	M
Haynes et al. (2019)	M	L	H	M	H	M	M	M
Jimenez-Olmedo et al. (2022)	H	M	H	M	M	H	M	M
Patiño-Palma et al. (2022)	H	H	H	M	M	H	M	M
Plakoutsis et al. (2023)	H	M	H	M	H	H	M	M
Soares et al. (2023)	M	M	H	M	M	M	M	M
Stanton et al. (2017)	H	M	H	M	H	H	H	M
Yingling et al. (2018)	H	H	H	M	L	M	M	M

*L* low quality, *M* moderate quality, *H* high quality, *I* inadequate.

### Meta-analysis results

#### Heterogeneity and publication bias outputs

The heterogeneity statistics and publication bias were assessed for three main categories: Mean differences, Reliability analysis (ICC values), and Validity analysis (r values). For mean differences, the Cochran Q statistic was 46.67 (p < 0.001), with an I^2^ value of 70.0% and a *tau*^2^ of 0.062. Egger’s test for publication bias was not significant (p = 0.860). For reliability analysis, the Cochran Q statistic was notably high at 512.5 (p < 0.001). The I^2^ value was 96.5%, and *tau*^2^ was 0.397. Egger’s test indicated a p-value of 0.156. For validity analysis, the Cochran Q statistic was 959.2 (p < 0.001), with an I^2^ value of 98.3% and a *tau*^2^ of 0.864. Egger's test showed a p-value of 0.436. High I^2^ values, such as those observed in the Reliability and Validity analyses, indicate substantial heterogeneity among the included studies. An I^2^ value above 75% is generally considered to represent considerable heterogeneity. This suggests that the observed variations in effect sizes are not solely due to sampling error but may be attributed to other factors, such as methodological differences or population characteristics among the studies (Table 3).

**Table 3:** Summary statistics related to the heterogeneity and publication bias.

Validity and reliability analyses	Heterogeneity statistics				Publication bias	
	Q	*p*	I^2^	*Tau*^2^	Egger	*p*
Mean differences	46.67	< 0.001	70.0	0.062	0.29	0.860
Reliability analysis (ICC values)	512.5	< 0.001	96.5	0.397	4.61	0.156
Validity analysis (r values)	959.2	< 0.001	98.3	0.864	4.16	0.436

*ICC* intraclass correlation coefficients. *Q* Cochran Q statistic for homogeneity test, I^2^: the proportion of total variation caused by heterogeneity rather than within‐study sampling error (%), *Tau*^2^: the variance in true effect sizes observed in different studies, Egger: Egger’s regression test.

#### Validity outputs

**Figure 2:**
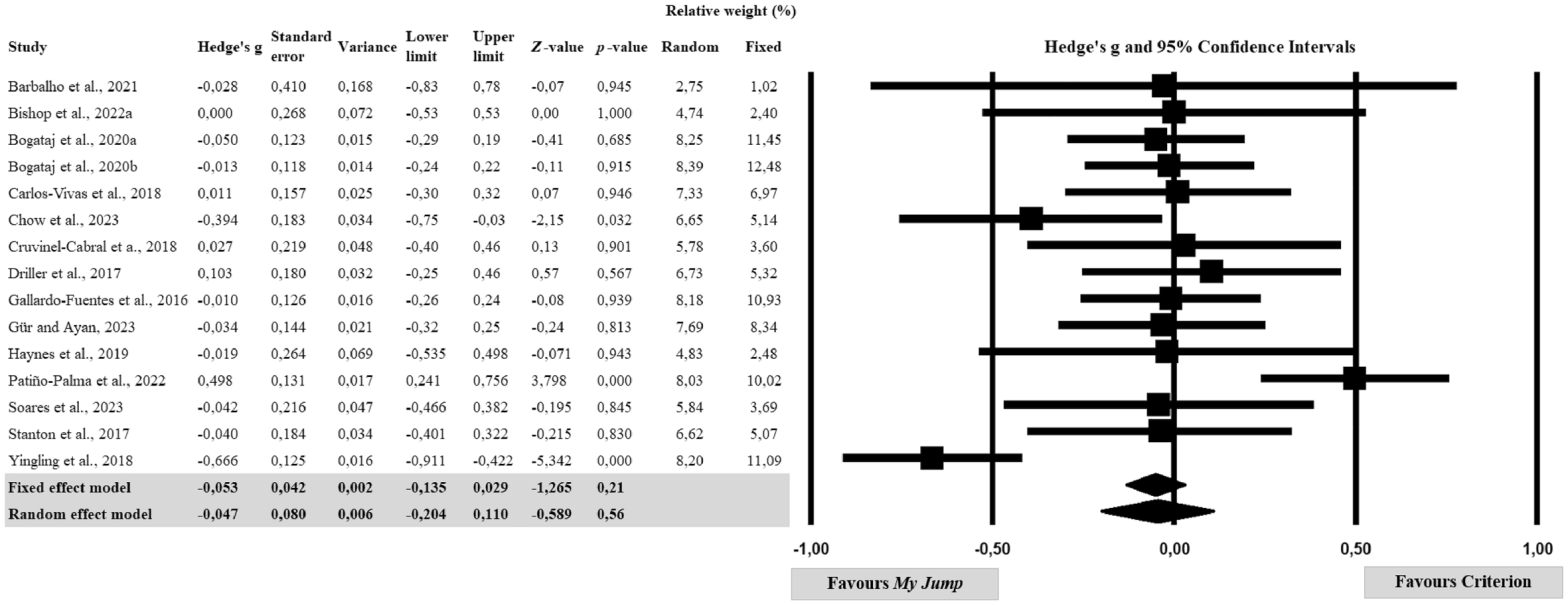
Forest plot of differences between *My Jump* and related criterion measures. Values shown are Hedge’s g with 95% confidence intervals. The size of the plotted squares represents the relative weight of the study.

The meta-analysis conducted for the agreement between raw scores showed that there was no significant difference between *My Jump* and the criteria (Hedge’s g = − 0.047; *p* = 0.21, Fig. 2). Further analyses showed a significant heterogeneity (Q = 46.67; *p* < 0.001; *tau*^2^ = 0.062), with an *I*^2^ value indicating 70.0% of effect size variance accounted for across the individual studies (Table 3). Because of the significant heterogeneity, the pooled effect size was conducted according to the random-effects model. Additionally, the risk of publication bias was explored using funnel plot symmetry and confirmed using the extended Egger’s test (Table 3). Egger’s test did not show any potential asymmetry (*p* = 0.860).

The sub-analysis for CMJ included 13 studies. The fixed effect model yielded a Hedge’s g value of − 0.060 (95% CI − 0.151 to 0.032, p = 0.202), while the random effect model indicated a Hedge's g value of − 0.047 (95% CI − 0.233 to 0.139, p = 0.622). The studies exhibited varying degrees of relative weight, ranging from 3.04 to 14.07%. In the sub-analysis for Squat SQJ, three studies were included. The fixed and random effect models both showed a Hedge’s g value of − 0.020 (95% CI − 0.257 to 0.217, p = 0.869). The studies in this category had relative weights of 31.38%, 32.83%, and 35.78%. The sub-analysis for DJ comprised four studies. Both the fixed and random effect models indicated a Hedge’s g value of − 0.034 (95% CI − 0.295 to 0.226, p = 0.795). The relative weights for the studies in this sub-analysis ranged from 10.50 to 37.79% (Table 4).

**Table 4:** Sub-validity analyses for vertical jump types based on Hedge’s g values.

Study	Hedge’s g	Standard error	Variance	Lower limit	Upper limit	*Z*-value	*p*-value	Relative weight (%)	
								Random	Fixed
Sub-analysis for countermovement jump (CMJ)									
Bishop et al. (2022a)	0.000	0.268	0.072	– 0.53	0.53	0.00	1.000	5.79	3.04
Bogataj et al. (2020a)	– 0.049	0.150	0.023	– 0.34	0.24	– 0.33	0.742	8.49	9.71
Bogataj et al. (2020b)	– 0.028	0.144	0.021	– 0.31	0.25	– 0.20	0.845	8.64	10.59
Carlos–Vivas et al. (2018)	0.011	0.157	0.025	– 0.30	0.32	0.07	0.946	8.31	8.84
Chow et al. (2023)	– 0.394	0.183	0.034	– 0.75	–0.03	– 2.15	0.032	7.69	6.52
Cruvinel-Cabral et al. (2018)	0.027	0.219	0.048	– 0.40	0.46	0.13	0.901	6.85	4.57
Driller et al. (2017)	0.103	0.180	0.032	– 0.25	0.46	0.57	0.567	7.76	6.75
Gallardo-Fuentes et al. (2016)	– 0.005	0.216	0.047	– 0.43	0.42	– 0.02	0.981	6.91	4.68
Gür and Ayan, (2023)	– 0.034	0.144	0.021	– 0.32	0.25	– 0.24	0.813	8.64	10.59
Patiño-Palma et al. (2022)	0.498	0.131	0.017	0.24	0.76	3.80	0.000	8.94	12.71
Soares et al. (2023)	– 0.042	0.216	0.047	– 0.466	0.382	– 0.195	0.845	6.91	4.68
Stanton et al. (2017)	0.026	0.259	0.067	– 0.482	0.534	0.099	0.921	5.97	3.26
Yingling et al. (2018)	– 0.666	0.125	0.016	– 0.911	–0.422	– 5.342	0.000	9.09	14.07
Fixed effect model	– 0.060	0.047	0.002	– 0.151	0.032	– 1.276	0.202		
Random effect model	– 0.047	0.095	0.009	– 0.233	0.139	– 0.494	0.622		
Sub-analysis for squat jump (SQJ)									
Bogataj et al. (2020a)	– 0.064	0.211	0.045	– 0.48	0.35	– 0.30	0.760	32.83	32.83
Bogataj et al. (2020b)	0.023	0.202	0.041	– 0.37	0.42	0.11	0.909	35.78	35.78
Gallardo-Fuentes et al. (2016)	– 0.022	0.216	0.047	– 0.45	0.40	– 0.10	0.917	31.38	31.38
Fixed effect model	– 0.020	0.121	0.015	– 0.257	0.217	– 0.165	0.869		
Random effect model	– 0.020	0.121	0.015	– 0.257	0.217	– 0.165	0.869		
Sub-analysis for drop jump (DJ)									
Barbalho et al. (2021)	– 0.028	0.410	0.168	– 0.83	0.78	– 0.07	0.945	10.50	10.50
Gallardo-Fuentes et al. (2016)	0.000	0.216	0.047	– 0.42	0.42	0.00	1.000	37.79	37.79
Haynes et al. (2019)	– 0.019	0.264	0.069	– 0.54	0.50	– 0.07	0.943	25.43	25.43
Stanton et al. (2017)	– 0.102	0.259	0.067	– 0.61	0.41	– 0.39	0.695	26.28	26.28
Fixed effect model	– 0.034	0.133	0.018	– 0.295	0.226	– 0.259	0.795		
Random effect model	– 0.034	0.133	0.018	– 0.295	0.226	– 0.259	0.795		

The sub-analysis focused on studies utilizing Force Plates included six studies. The fixed and random effect models both indicated a Hedge’s g value of 0.015 (95% CI − 0.154 to 0.184, p = 0.863). The relative weights for the studies in this sub-analysis ranged from 4.41 to 29.95%. In the sub-analysis focused on studies not utilizing Force Plates, nine studies were included. The fixed effect model showed a Hedge’s g value of − 0.073 (95% CI − 0.166 to 0.020, p = 0.124), while the random effect model indicated a Hedge’s g value of − 0.075 (95% CI − 0.302 to 0.151, p = 0.515). The studies in this category had relative weights ranging from 4.69 to 16.27% (Table 5).

**Table 5:** Sub-validity analyses for the criterion device based on Hedge’s g values.

Study	Hedge’s g	Standard error	Variance	Lower limit	Upper limit	*Z*-value	*p*-value	Relative weight (%)	
								Random	Fixed
Sub-analysis focused on studies utilizing force plates for criterion measurement									
Barbalho et al. (2021)	– 0.028	0.410	0.168	– 0.83	0.78	– 0.07	0.945	4.41	4.41
Bishop et al. (2022a)	0.000	0.268	0.072	– 0.53	0.53	0.00	1.000	10.31	10.31
Carlos-Vivas et al. (2018)	0.011	0.157	0.025	– 0.30	0.32	0.07	0.946	29.95	29.95
Driller et al. (2017)	0.103	0.180	0.032	– 0.25	0.46	0.57	0.567	22.87	22.87
Haynes et al. (2019)	– 0.019	0.264	0.069	– 0.54	0.50	– 0.07	0.943	10.68	10.68
Stanton et al. (2017)	– 0.040	0.184	0.034	– 0.40	0.32	– 0.22	0.830	21.79	21.79
Fixed effect model	0.015	0.086	0.007	– 0.154	0.184	0.173	0.863		
Random effect model	0.015	0.086	0.007	– 0.154	0.184	0.173	0.863		
Sub-analysis focused on studies not utilizing force plates for criterion measurement									
Bogataj et al. (2020a)	– 0.050	0.123	0.015	– 0.29	0.19	– 0.41	0.685	11.98	14.91
Bogataj et al. (2020b)	– 0.013	0.118	0.014	– 0.24	0.22	– 0.11	0.915	12.12	16.27
Chow et al. (2023)	– 0.394	0.183	0.034	– 0.75	– 0.03	– 2.15	0.032	10.28	6.70
Cruvinel-Cabral et al. (2018)	0.027	0.219	0.048	– 0.40	0.46	0.13	0.901	9.26	4.69
Gallardo-Fuentes et al. (2016)	– 0.010	0.126	0.016	– 0.26	0.24	– 0.08	0.939	11.91	14.24
Gür and Ayan (2023)	– 0.034	0.144	0.021	– 0.32	0.25	– 0.24	0.813	11.41	10.87
Patiño-Palma et al. (2022)	0.498	0.131	0.017	0.24	0.76	3.80	0.000	11.76	13.05
Soares et al. (2023)	– 0.042	0.216	0.047	– 0.47	0.38	– 0.20	0.845	9.34	4.81
Yingling et al. (2018)	– 0.666	0.125	0.016	– 0.91	– 0.42	– 5.34	0.000	11.93	14.45
Fixed effect model	– 0.073	0.047	0.002	– 0.166	0.020	– 1.538	0.124		
Random effect model	– 0.075	0.116	0.013	– 0.302	0.151	– 0.651	0.515		

**Figure 3:**
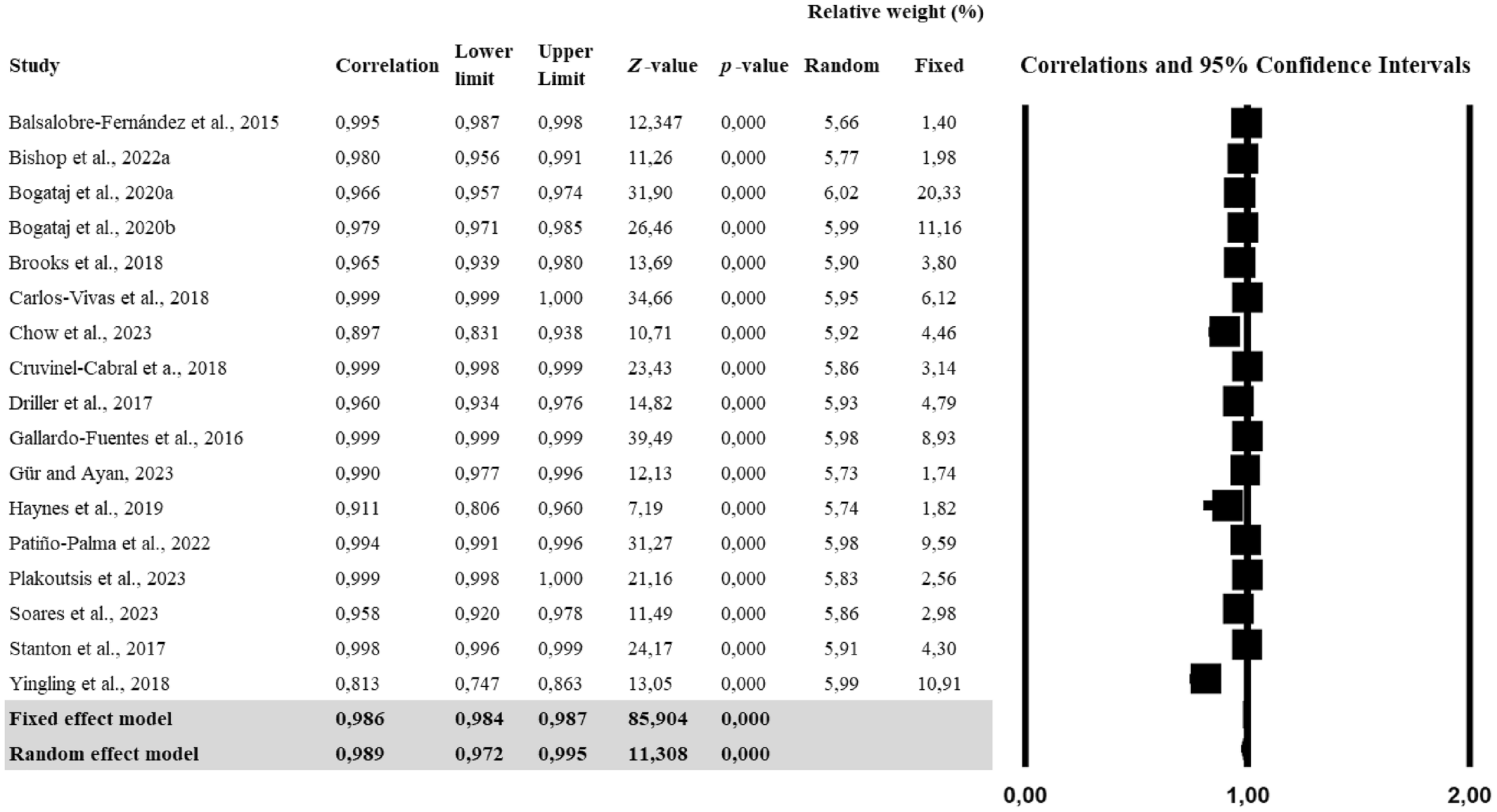
Forest plot for correlations between *My Jump* and related criterion measures. Values shown are correlation coefficient with 95% confidence intervals. The size of the plotted squares represents the relative weight of the study.

The meta-analysis conducted for identifying the consistency of the rankings within-group showed a high correlation (r = 0.989) between *My Jump* and criteria while individual studies reported correlations ranging from 0.813 to 0.999 (Fig. 3). Further analyses showed a significant heterogeneity (Q = 959.2; *p* < 0.001; *tau*^2^ = 0.864), with an *I*^2^ value indicating 98.3% of effect size variance accounted for across the individual studies (Table 3). The risk of publication bias was explored using funnel plot symmetry and confirmed using the extended Egger’s test (Table 3). Additionally, the risk of publication bias was explored using funnel plot symmetry and confirmed using the extended Egger’s test (Table 3). Egger's test did not show any potential asymmetry (p = 0.436).

For CMJ, the fixed effect model showed a correlation of 0.990 (95% CI 0.988–0.991, p < 0.001), while the random effect model indicated a correlation of 0.992 (95% CI 0.981–0.996, p < 0.001). For SQJ, the fixed effect model indicated a correlation of 0.984 (95% CI 0.978–0.988, p < 0.001), and the random effect model showed a correlation of 0.989 (95% CI 0.913–0.999, p < 0.001). For DJ, the fixed effect model revealed a correlation of 0.996 (95% CI 0.994–0.997, p < 0.001), and the random effect model indicated a correlation of 0.994 (95% CI 0.923–1.000, p < 0.001). These results suggest that the different types of jumps are highly correlated with the criterion measures, indicating strong validity across the board (Table 6).

**Table 6:** Sub-validity analyses for vertical jump types based on correlation values.

Study	Correlation	Lower limit	Upper Limit	*Z*-value	*p*-value	Relative weight (%)	
						Random	Fixed
Sub-analysis for countermovement jump (CMJ)							
Balsalobre-Fernández et al. (2015)	0.995	0.987	0.998	12.347	0.000	6.36	2.05
Bishop et al. (2022a)	0.980	0.956	0.991	11.26	0.000	6.52	2.89
Bogataj et al. (2020a)	0.969	0.958	0.977	26.51	0.000	6.87	19.74
Bogataj et al. (2020b)	0.983	0.974	0.988	22.48	0.000	6.82	10.83
Brooks et al. (2018)	0.965	0.939	0.980	13.69	0.000	6.71	5.54
Carlos-Vivas et al. (2018)	0.999	0.999	1.000	34.66	0.000	6.79	8.90
Chow et al. (2023)	0.897	0.831	0.938	10.71	0.000	6.74	6.50
Cruvinel-Cabral et al. (2018)	0.999	0.998	0.999	23.43	0.000	6.67	4.57
Driller et al. (2017)	0.960	0.934	0.976	14.82	0.000	6.75	6.98
Gallardo-Fuentes et al. (2016)	0.999	0.998	0.999	22.80	0.000	6.65	4.33
Gür and Ayan (2023)	0.990	0.977	0.996	12.13	0.000	6.46	2.53
Patiño-Palma et al. (2022)	0.994	0.991	0.996	31.27	0.000	6.84	13.96
Plakoutsis et al. (2023)	0.999	0.998	1.000	21.16	0.000	6.61	3.73
Soares et al. (2023)	0.958	0.920	0.978	11.49	0.000	6.65	4.33
Stanton et al. (2017)	0.997	0.994	0.999	16.57	0.000	6.55	3.13
Fixed effect model	0.990	0.988	0.991	75.834	0.000		
Random effect model	0.992	0.981	0.996	12.865	0.000		
Sub-analysis for squat jump (SJ)							
Bogataj et al. (2020a)	0.961	0.941	0.975	17.767	0.000	33.65	50.31
Bogataj et al. (2020b)	0.970	0.947	0.983	14.04	0.000	33.28	27.61
Gallardo-Fuentes et al. (2016)	0.999	0.998	0.999	22.80	0.000	33.07	22.09
Fixed effect model	0.984	0.978	0.988	30.692	0.000		
Random effect model	0.989	0.913	0.999	4.786	0.000		
Sub-analysis for drop jump (DJ)							
Gallardo-Fuentes et al. (2016)	0.999	0.998	0.999	22.801	0.000	33.56	42.86
Haynes et al. (2019)	0.911	0.806	0.960	7.19	0.000	33.13	26.19
Stanton et al. (2017)	0.998	0.996	0.999	17.61	0.000	33.30	30.95
Fixed effect model	0.996	0.994	0.997	28.404	0.000		
Random effect model	0.994	0.923	1.000	4.343	0.000		

The correlation analyses were conducted to evaluate the validity of different criterion measures, specifically force plates, and non-force plates, in assessing jump performance. The fixed effect model for studies using force plates showed a correlation of 0.994 (95% CI 0.992–0.995, p < 0.001), while the random effect model indicated a correlation of 0.992 (95% CI 0.968–0.998, p < 0.001). On the other hand, for studies not using force plates, the fixed effect model revealed a correlation of 0.981 (95% CI 0.978–0.983, p < 0.001), and the random effect model showed a correlation of 0.985 (95% CI 0.951–0.995, p < 0.001). These results highlight the robustness and high validity of both types of criterion measures in assessing different types of jumps, as evidenced by the consistently high correlations across studies (Table 7).

**Table 7:** Sub-validity analyses for criterion device based on correlation values.

Study	Correlation	Lower limit	Upper Limit	*Z*-value	*p*-value	Relative weight (%)	
						Random	Fixed
Sub-analysis focused on studies utilizing force plates for criterion measurement							
Balsalobre-Fernández et al. (2015)	0.995	0.987	0.998	12.347	0.000	12.16	5.25
Bishop et al. (2022a)	0.980	0.956	0.991	11.26	0.000	12.37	7.41
Brooks et al. (2018)	0.965	0.939	0.980	13.69	0.000	12.62	14.20
Carlos-Vivas et al. (2018)	0.999	0.999	1.000	34.66	0.000	12.73	22.84
Driller et al. (2017)	0.960	0.934	0.976	14.82	0.000	12.68	17.90
Haynes et al. (2019)	0.911	0.806	0.960	7.19	0.000	12.32	6.79
Plakoutsis et al. (2023)	0.999	0.998	1.000	21.16	0.000	12.48	9.57
Stanton et al. (2017)	0.998	0.996	0.999	24.17	0.000	12.65	16.05
Fixed effect model	0.994	0.992	0.995	51.984	0.000		
Random effect model	0.992	0.968	0.998	7.843	0.000		
Sub-analysis focused on studies not utilizing force plates for criterion measurement							
Bogataj et al. (2020a)	0.966	0.957	0.974	31.900	0.000	11.29	
Bogataj et al. (2020b)	0.979	0.971	0.985	26.46	0.000	11.24	
Chow et al. (2023)	0.897	0.831	0.938	10.71	0.000	11.09	
Cruvinel-Cabral et al. (2018)	0.999	0.998	0.999	23.43	0.000	10.99	
Gallardo-Fuentes et al. (2016)	0.999	0.999	0.999	39.49	0.000	11.22	
Gür and Ayan (2023)	0.990	0.977	0.996	12.13	0.000	10.72	
Patiño-Palma et al. (2022)	0.994	0.991	0.996	31.27	0.000	11.23	
Soares et al. (2023)	0.958	0.920	0.978	11.49	0.000	10.97	
Yingling et al. (2018)	0.813	0.747	0.863	13.05	0.000	11.24	
Fixed effect model	0.981	0.978	0.983	68.954	0.000		
Random effect model	0.985	0.951	0.995	8.019	0.000		

#### Reliability outputs

**Figure 4:**
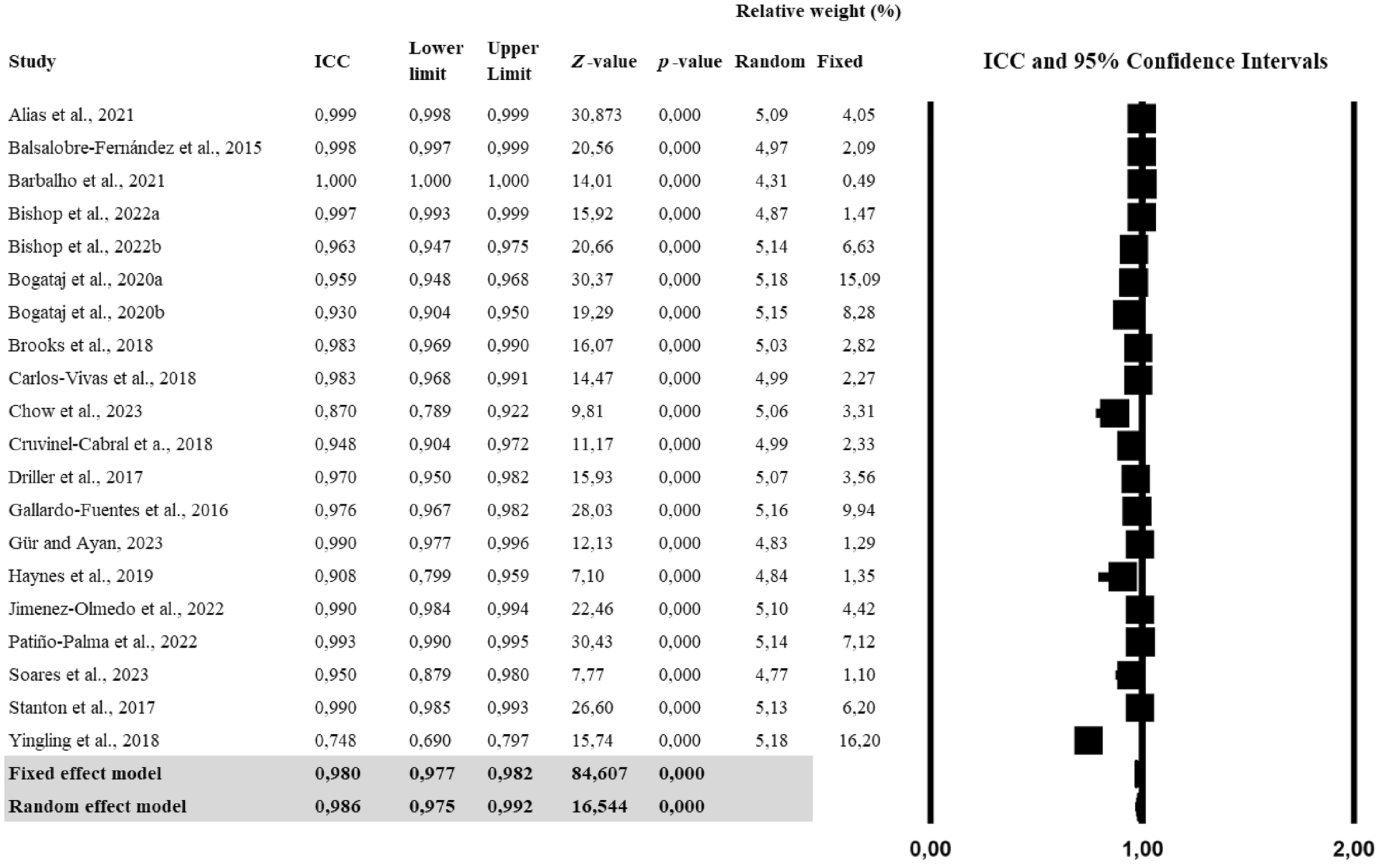
Forest plot for ICCs of *My Jump* measures. Values shown are ICC with 95% confidence intervals. The size of the plotted squares represents the relative weight of the study. *ICC* intraclass correlation coefficients.

The meta-analysis conducted for identifying the reliability of the *My Jump* smartphone application showed nearly perfect reliability scores (r = 0.986) while individual studies reported correlations ranging from 0.748 to 1.0 (Fig. 4). Further analyses showed a significant heterogeneity (Q = 512.5; *p* < 0.001; *tau*^2^ = 0.397), with an I^2^ value indicating 96.5% of effect size variance accounted for across the individual studies (Table 3). The risk of publication bias was explored using funnel plot symmetry and confirmed using the extended Egger’s test (Table 3). Egger’s test did not show any potential asymmetry (*p* = 0.386).

The ICC was used to assess the reliability of different types of jumps, including CMJ, SQJ, and DJ. For CMJ, the fixed effect model showed an ICC of 0.969 (95% CI 0.965–0.972, p < 0.001), and the random effect model indicated an ICC of 0.982 (95% CI 0.961–0.992, p < 0.001). For SJ, the fixed effect model revealed an ICC of 0.965 (95% CI 0.953–0.974, p < 0.001), and the random effect model showed an ICC of 0.961 (95% CI 0.889–0.987, p < 0.001). In the case of DJ, the fixed effect model indicated an ICC of 0.972 (95% CI 0.964–0.979, p < 0.001), while the random effect model showed an ICC of 0.987 (95% CI 0.940–0.997, p < 0.001). These results suggest that the methods used for assessing different types of jumps are highly reliable, as evidenced by the consistently high ICCs across studies (Table 8).

**Table 8:** Sub-reliability analyses for vertical jump types based on correlation values.

Study	ICC	Lower limit	Upper Limit	*Z*-value	*p*-value	Relative weight (%)	
						Random	Fixed
Sub-analysis for countermovement jump (CMJ)							
Alias et al. (2021)	0.999	0.998	0.999	30.873	0.000	5.61	5.23
Balsalobre-Fernández et al. (2015)	0.998	0.997	0.999	20.56	0.000	5.50	2.69
Bishop et al. (2022a)	0.997	0.993	0.999	15.92	0.000	5.41	1.90
Bishop et al. (2022b)	0.954	0.923	0.973	13.79	0.000	5.58	4.28
Bogataj et al. (2020a)	0.953	0.936	0.965	23.80	0.000	5.68	13.00
Bogataj et al. (2020b)	0.947	0.921	0.965	17.10	0.000	5.64	7.13
Brooks et al. (2018)	0.983	0.969	0.990	16.07	0.000	5.56	3.65
Carlos-Vivas et al. (2018)	0.983	0.968	0.991	14.47	0.000	5.52	2.93
Chow et al. (2023)	0.870	0.789	0.922	9.81	0.000	5.58	4.28
Cruvinel-Cabral et al. (2018	0.948	0.904	0.972	11.17	0.000	5.52	3.01
Driller et al. (2017)	0.970	0.950	0.982	15.93	0.000	5.59	4.60
Gallardo-Fuentes et al. (2016)	0.990	0.983	0.994	19.45	0.000	5.58	4.28
Gür and Ayan (2023)	0.990	0.977	0.996	12.13	0.000	5.36	1.66
Jimenez-Olmedo et al. (2022)	0.990	0.984	0.994	22.46	0.000	5.62	5.71
Patiño-Palma et al. (2022)	0.993	0.990	0.995	30.43	0.000	5.66	9.19
Soares et al. (2023)	0.950	0.879	0.980	7.77	0.000	5.31	1.43
Stanton et al. (2017)	0.990	0.983	0.994	19.09	0.000	5.58	4.12
Yingling et al. (2018)	0.748	0.690	0.797	15.74	0.000	5.70	20.92
Fixed effect model	0.969	0.965	0.972	73.738	0.000		
Random effect model	0.982	0.961	0.992	11.761	0.000		
Sub-analysis for squat jump (SJ)							
Bogataj et al. (2020a)	0.970	0.954	0.980	18.947	0.000	34.11	45.30
Bogataj et al. (2020b)	0.880	0.795	0.931	9.23	0.000	32.69	24.86
Gallardo-Fuentes et al. (2016)	0.984	0.973	0.991	17.74	0.000	33.20	29.83
Fixed effect model	0.965	0.953	0.974	27.043	0.000		
Random effect model	0.961	0.889	0.987	7.004	0.000		
Sub-analysis for drop jump (DJ)							
Barbalho et al. (2021)	1.000	1.000	1.000	14.006	0.000	18.05	4.28
Bishop et al. (2022b)	0.970	0.950	0.983	15.427	0.000	20.68	28.88
Gallardo-Fuentes et al. (2016)	0.913	0.856	0.948	11.358	0.000	20.68	28.88
Haynes et al. (2019)	0.908	0.799	0.959	7.10	0.000	19.95	11.76
Stanton et al. (2017)	0.990	0.983	0.994	18.53	0.000	20.63	26.20
Fixed effect model	0.972	0.964	0.979	29.210	0.000		
Random effect model	0.987	0.940	0.997	6.424	0.000		

For studies that utilized Force Plates for Criterion Measurement, the fixed effect model showed an ICC of 0.989 (95% CI 0.986–0.991, p < 0.001), and the random effect model indicated an ICC of 0.993 (95% CI 0.981–0.997, p < 0.001). For studies that did not utilize Force Plates for Criterion Measurement, the fixed effect model showed an ICC of 0.960 (95% CI 0.955–0.964, p < 0.001), and the random effect model indicated an ICC of 0.972 (95% CI 0.936–0.988, p < 0.001). These findings suggest that both methods, whether utilizing force plates or not, are highly reliable for the measurements they aim to assess, as evidenced by the consistently high ICCs across studies (Table 9).

**Table 9:** Sub-reliability analyses for criterion device based on correlation values.

Study	ICC	Lower limit	Upper Limit	*Z*-value	*p*-value	Random	Fixed
Sub-analysis focused on studies utilizing force plates for criterion measurement							
Balsalobre-Fernández et al. (2015)	0.998	0.997	0.999	20.556	0.000	12.71	10.30
Barbalho et al. (2021)	1.000	1.000	1.000	14.01	0.000	10.60	2.42
Bishop et al. (2022a)	0.997	0.993	0.999	15.92	0.000	12.40	7.27
Brooks et al. (2018)	0.983	0.969	0.990	16.07	0.000	12.92	13.94
Carlos-Vivas et al. (2018)	0.983	0.968	0.991	14.47	0.000	12.78	11.21
Driller et al. (2017)	0.970	0.950	0.982	15.93	0.000	13.04	17.58
Haynes et al. (2019)	0.908	0.799	0.959	7.10	0.000	12.30	6.67
Stanton et al. (2017)	0.990	0.985	0.993	26.60	0.000	13.25	30.61
Fixed effect model	0.989	0.986	0.991	47.149	0.000		
Random effect model	0.993	0.981	0.997	11.337	0.000		
Sub-analysis focused on studies not utilizing force plates for criterion measurement							
Alias et al. (2021)	0.999	0.998	0.999	30.873	0.000	8.37	5.08
Bishop et al. (2022b)	0.963	0.947	0.975	20.66	0.000	8.45	8.31
Bogataj et al. (2020a)	0.959	0.948	0.968	30.37	0.000	8.53	18.92
Bogataj et al. (2020b)	0.930	0.904	0.950	19.29	0.000	8.48	10.38
Chow et al. (2023)	0.870	0.789	0.922	9.81	0.000	8.32	4.15
Cruvinel-Cabral et al. (2018)	0.948	0.904	0.972	11.17	0.000	8.21	2.92
Gallardo-Fuentes et al. (2016)	0.976	0.967	0.982	28.03	0.000	8.50	12.46
Gür and Ayan (2023)	0.990	0.977	0.996	12.13	0.000	7.93	1.62
Jimenez-Olmedo et al. (2022)	0.990	0.984	0.994	22.46	0.000	8.39	5.54
Patiño-Palma et al. (2022)	0.993	0.990	0.995	30.43	0.000	8.46	8.92
Soares et al. (2023)	0.950	0.879	0.980	7.77	0.000	7.83	1.38
Yingling et al. (2018)	0.748	0.690	0.797	15.74	0.000	8.53	20.31
Fixed effect model	0.960	0.955	0.964	70.066	0.000		
Random effect model	0.972	0.936	0.988	9.656	0.000		

The sub-analysis revealed high levels of reliability across different contexts. For inter-rater reliability, the fixed effect model showed an ICC of 0.993 (95% CI 0.991–0.994, p < 0.001), and the random effect model indicated an ICC of 0.996 (95% CI 0.987–0.999, p < 0.001). In terms of intra-rater reliability, the fixed effect model revealed an ICC of 0.995 (95% CI 0.993–0.996, p < 0.001), and the random effect model showed an ICC of 0.997 (95% CI 0.990–0.999, p < 0.001). For inter-session reliability, both the fixed and random effect models showed an ICC of 0.970 (95% CI 0.954–0.981, p < 0.001). Lastly, within session/device reliability had a fixed effect model ICC of 0.946 (95% CI 0.940–0.952, p < 0.001) and a random effect model ICC of 0.973 (95% CI 0.945–0.987, p < 0.001). These consistently high ICCs across studies suggest that the methods used for assessing different aspects of reliability are highly robust (Table 10).

**Table 10:** Sub-reliability analyses for reliability types based on correlation values.

Study	ICC	Lower limit	Upper Limit	*Z*-value	*p*-value	Relative weight (%)	
						Random	Fixed
Sub-analysis for inter-rater reliability							
Alias et al. (2021)	0.999	0.998	1.000	17.825	0.000	14.20	7.53
Balsalobre-Fernández et al. (2015)	0.998	0.997	0.999	20.56	0.000	14.61	11.64
Barbalho et al. (2021)	1.000	1.000	1.000	14.01	0.000	12.47	2.74
Driller et al. (2017)	0.970	0.950	0.982	15.93	0.000	14.94	19.86
Jimenez-Olmedo et al. (2022)	0.990	0.981	0.995	15.88	0.000	14.65	12.33
Patiño-Palma et al. (2022)	0.993	0.990	0.995	30.43	0.000	15.18	39.73
Soares et al. (2023)	0.950	0.879	0.980	7.77	0.000	13.96	6.16
Fixed effect model	0.993	0.991	0.994	48.014	0.000		
Random effect model	0.996	0.987	0.999	10.712	0.000		
Sub-analysis for intra-rater reliability							
Alias et al. (2021)	0.999	0.998	1.000	17.825	0.000	20.17	15.94
Barbalho et al. (2021)	1.000	1.000	1.000	14.01	0.000	17.68	5.80
Jimenez-Olmedo et al. (2022)	0.990	0.981	0.995	15.88	0.000	20.82	26.09
Brooks et al. (2018)	0.990	0.977	0.996	12.69	0.000	20.24	16.67
Stanton et al. (2017)	0.990	0.983	0.994	18.53	0.000	21.10	35.51
Fixed effect model	0.995	0.993	0.996	34.821	0.000		
Random effect model	0.997	0.990	0.999	9.726	0.000		
Sub-analysis for inter-session reliability							
Brooks et al. (2018)	0.970	0.933	0.987	10.034	0.000	29.87	29.87
Gallardo-Fuentes et al. (2016)	0.970	0.949	0.982	15.38	0.000	70.13	70.13
Fixed effect model	0.970	0.954	0.981	18.361	0.000		
Random effect model	0.970	0.954	0.981	18.361	0.000		
Sub-analysis for within session/device reliability							
Alias et al. (2021)	0.999	0.998	1.000	17.825	0.000	7.33	1.95
Gallardo-Fuentes et al. (2016)	0.978	0.969	0.985	23.45	0.000	7.93	9.55
Stanton et al. (2017)	0.990	0.983	0.994	19.09	0.000	7.75	4.60
Bishop et al. (2022a)	0.997	0.993	0.999	15.92	0.000	7.39	2.12
Bishop et al. (2022b)	0.963	0.947	0.975	20.66	0.000	7.93	9.55
Bogataj et al. (2020a)	0.959	0.948	0.968	30.37	0.000	8.02	21.75
Bogataj et al. (2020b)	0.930	0.904	0.950	19.29	0.000	7.96	11.94
Carlos-Vivas et al. (2018)	0.983	0.968	0.991	14.47	0.000	7.62	3.27
Chow et al. (2023)	0.870	0.789	0.922	9.81	0.000	7.77	4.77
Cruvinel-Cabral et a. (2018)	0.948	0.904	0.972	11.17	0.000	7.64	3.36
Gür and Ayan, (2023)	0.990	0.977	0.996	12.13	0.000	7.30	1.86
Haynes et al. (2019)	0.908	0.799	0.959	7.10	0.000	7.33	1.95
Yingling et al. (2018)	0.748	0.690	0.797	15.74	0.000	8.03	23.34
Fixed effect model	0.946	0.940	0.952	60.414	0.000		
Random effect model	0.973	0.945	0.987	11.489	0.000		

## Discussion

In this systematic review and meta-analysis, the validity and reliability findings of the *My Jump* smartphone application, which is designed to measure vertical jump performance, were summarized using meta-analytical methods. This review summarized the findings of 21 studies consisting of 839 accumulated participants. Overall methodological quality assessment for individual studies included in this meta-analysis was considered as moderate-to-high quality. Further analyses showed significant heterogeneity scores; thus, the pooled calculations were interpreted according to the random-effects model. For validity, meta-analysis results revealed that there was a raw score agreement between *My Jump* and the criterion measures, based on nonsignificant Hedge's g values as well as a high consistency of the within-group rankings, based on the pooled correlation result. For reliability, our meta-analysis showed near-perfect reliability for *My Jump*, based on the pooled ICC value. Additionally, sub-analyses suggested that the results were robust across different types of jumps, reference devices used, and types of reliability.

In fact, unlike the usual study designs that investigate the validity and the test–retest reliability of athletic performance measures, validity, and reliability analyses of the *My Jump* application can be completed without the need for re-testing^14,45,46^. As the participant performs a vertical jump once on a platform, the nature of which is accepted as the criterion (force plate, mat, photocell sensors, etc.), a video can be simultaneously recorded^12,13,47^. Thus, possible biases can be attributed to other factors not regarding participants. For example, because take-off and landing points are manually marked, minor variations are likely when a rater measures the same vertical jump performance consecutively. Or, in a video recording measuring a single jump performance, two raters may mark take-off and landing points differently. However, the *My Jump* provides a very functional method to minimize these errors, as it offers the possibility to pause the video and play it frame by frame^12,13,47^. Additionally, the formula it uses (h = t^2^ × 1.22625)^48^ is equivalent to most criterion devices. In this case, the major handicap seems to be small variations that can arise from manually determining the take-off and landing points.

While the *My Jump* application relies on flight time to calculate jump height, force platforms are often considered the gold standard due in part to their ability to calculate jump height based on the impulse-momentum theorem, which takes into account the total force applied during the jump and the duration of this force, providing a more holistic assessment^15,16^. However, it's worth noting that flight time-based calculations are also commonly used in force platforms. In fact, in the initial study introducing the *My Jump*, a force platform was used as a reference device, and it too employed a flight time-based methodology for the sake of comparison^47^. This methodological overlap offers some advantages. For instance, strategies that could artificially lengthen flight time are also applicable to force platform measurements based on flight time^15^. Therefore, in scenarios where the device rather than the method serves as the reference for jump performance, My Jump appears to be a viable alternative. The primary objective of our study is to test whether My Jump can serve as an alternative to more expensive and less portable devices. Our findings suggest that My Jump can be reliably used for practical applications such as ranking the jump performance of members within a group or tracking an athlete's jump performance over time, provided that the same methodology is consistently applied.

The importance of a high sampling rate is indeed critical for the My Jump app, which utilizes video recordings to calculate jump metrics^3,19,47^. In the study introducing this smartphone application, an iPhone 5 s was employed, featuring a 120 Hz high-speed camera at a quality of 720p, deemed adequate for such calculations^47^. Moreover, newer models of the device offer even more advanced capabilities^3,49,50^. When calculating jump height based on flight time, consider an average jump height of, for instance, 30 cm. The time an athlete would spend airborne for such a jump is approximately half a second. With a 120 Hz high-speed camera, this duration would translate into 60 frames (120 frames/second * 0.5 s = 60 frames). This high frame rate can allow for an accurate and reliable calculation of flight time, thus offering a valid measurement of jump height. What appears to be crucial is the adoption of a standardized procedure for selecting the frames where the jump starts and ends, ensuring consistency and reliability in measurements^1,51,52^.

Measurement errors related to instruments and raters in vertical jump assessments by smartphone applications can be caused to miss clinically crucial changes in performance^1,2,8^. However, while all of the studies^3,12–14,19,45–47,49,53–55^ that evaluated the validity and reliability of the *My Jump* included designs in which vertical jump performance was measured with two devices at the same time, no study had a comprehensive design containing the vertical jump height differences between two scores by the same rater and between the scores of two different raters, from the same video recording. Although some studies reported the comparison results related to differences between successive jumps performed several minutes apart or the differences between two test days^55,56^, possible errors in these designs can be attributed to the participants. For an application such as *My Jump*, in which data can be collected simultaneously along with the criterion device, it is more critical to focus on errors between raters, between devices, and between the same participant’s scores, rather than participant-sourced factors. Presenting a pooled reliability score using all reported ICC scores of original studies since they did not consistently provide ICC reports can be considered as a limitation for the present systematic review and meta-analysis.

Twenty studies^3,12–14,19,45–47,49–55,57–61^ using force plate, contact mat, and photocell system to examine the validity and reliability of the *My Jump* reported high (ICC > 0.80) reliability scores. However, one study^14^ compared vertical jump heights obtained from Vertec with the heights obtained from the *My Jump* and found the ICC score for absolute agreement to be 0.665. Although studies are showing that Vertec offers valid and reliable results^62,63^, considering the results of the other studies using more valid criterion methods, it seems highly likely that the inconsistency between the scores from two methods is related to the linear position transducers method used by Yingling et al.^14^. In addition, the fact that individual studies comprising participants that represent a wide range of the population, such as healthy adults, athletes, both men and women, children, and the elderly, strengthen the competence of this smartphone application to produce valid and reliable outputs. Consequently, the present systematic review and meta-analysis showed that the *My Jump* presented high agreement and consistency scores with the force plate, contact mat, and photocell systems as reference methods, demonstrating a pooled nearly perfect reliability score. In addition to its low-cost and simplicity, the *My Jump* smartphone application could be considered a valid and reliable method of assessing vertical jump height in various populations (Supplementary Information).

## Conclusions

This is the first investigation using meta-analytical methods to confirm the validity and reliability of the *My Jump* smartphone application to measure vertical jump heights. In terms of validity, meta-analysis results revealed that there was a raw score agreement between *My Jump* and the criterion measures, based on nonsignificant Hedge’s g values as well as a high consistency of the within-group rankings, based on the pooled correlation result. In terms of reliability, our present meta-analysis showed near-perfect reliability for *My Jump*, based on the pooled ICC value. Data from this systematic review and meta-analysis suggests that the *My Jump* can be used for assessing and monitoring vertical jump performance, which is a parameter included in global physical fitness test batteries and which provides information about the neuromuscular function and explosive power of the lower body. However, included studies mostly targeted on adults, only one study focused on children. More research need to be conducted on this population to precisely ensure the validity and reliability of *My Jump* smartphone application.

### Practical application

The findings of this systematic review and meta-analysis offer several practical implications for sports scientists, strength and conditioning practitioners, and coaches. The *My Jump* smartphone application provides a cost-effective and portable alternative to traditional laboratory equipment, making it accessible and convenient for teams or organizations with budget constraints. Its high validity and reliability make it a trustworthy tool for assessing an athlete's neuromuscular function and explosive power. While the application has been primarily validated in adult populations, its potential applicability across different age groups, including children, suggests a broader utility, although more research is needed in this area. The applications mobile-based platform allows for real-time monitoring and immediate feedback during training sessions, facilitating data-driven adjustments to training programs. Overall, the *My Jump* serves as a reliable and valid tool for accurately assessing vertical jump performance, offering a cost-effective and accessible means for data collection in various settings.

### Supplementary Information

Supplementary Information 1.

Supplementary Information 2.

## Data Availability

The datasets used and/or analyzed during the current study available from the corresponding author on reasonable request.
